# Use of ^99m^Tc 2-methoxyisobutyl isonitrile in minimally invasive radioguided surgery in patients with primary hyperparathyroidism: A narrative review of the current literature

**DOI:** 10.1002/jmrs.14

**Published:** 2013-06-03

**Authors:** Kristie A Denmeade, Chris Constable, Warren M Reed

**Affiliations:** 1 Nuclear Medicine and Ultrasound Department, Bankstown-Lidcombe HospitalBankstown, New South Wales, Australia; 2 Brain and Mind Research Institute, University of SydneyNew South Wales, Australia; 3 Discipline of Medical Radiation Sciences, Faculty of Health Sciences, The University of SydneyNew South Wales, Australia

**Keywords:** ^99m^Tc sestamibi, hyperparathyroidism, minimally invasive radioguided surgery, parathyroid adenoma, parathyroid scintigraphy, parathyroidectomy, primary hyperparathyroidism, radioguided

## Abstract

The use of technetium-99m 2-methoxyisobutyl isonitrile (^99m^Tc MIBI) for assistance in minimally invasive radioguided surgery (MIRS) is growing in popularity as a safe, effective, and proficient technique used for parathyroidectomy in primary hyperparathyroidism (PHPT) treatment. Previously, the preferred treatment for PHPT was bilateral neck exploration (BNE), a very invasive, costly, and lengthy procedure. However, as a large majority (80–85% of cases of PHPT) are attributed to a single parathyroid adenoma (PA), a simpler more direct technique such as MIRS is a far better option. The following article is an exploration of the current literature concerning varied protocols utilizing ^99m^Tc MIBI for assistance in MIRS for patients undergoing treatment of PHPT. This technique boasts many advantageous outcomes for patients suffering from PHPT. These include a reduction in cost, operating time, and patient recovery; less evidence of post-surgical hypocalcaemia, less pain, and complications; superior cosmetic results; same-day discharge; and the possibility of local anaesthesia which is particularly beneficial in elderly patients. Better outcomes for patients with deep or ectopic PAs, reduced intra-operative complications, and improved cosmetic outcomes for patients who have previously undergone thyroid and/or parathyroid surgery are also advantageous. Of the literature reviewed it was also found that no patients suffered any major surgical complications such as laryngeal nerve palsy or permanent hypoparathyroidism using ^99m^Tc MIBI for assistance in MIRS.

## Introduction

The parathyroids are very small glands normally found in the visceral space of the neck, posterior to the thyroid. While most people possess two superior and inferior parathyroid glands, people have been known to have only one, two, or three glands present. Normal parathyroid glands are around 6 mm in length, 3–4 mm in transverse diameter, and 1–2 mm in anteroposterior diameter. The glands weigh approximately 29.5 mg with an upper limit of 65 mg.^1^

Primary hyperparathyroidism (PHPT) occurs due to the overproduction of parathyroid hormone (PTH) in the body and is one of the most common endocrine disorders diagnosed throughout the world.^2^ Excess secretion of PTH results in hypercalcaemia and can be attributable to neurological, psychiatric, or muscular problems and brittle bones^3^; however, 50% or more of newly diagnosed cases of PHPT may be completely asymptomatic, and are detected by commonly used multi-channel blood screening tests.^2^,^4^ In the majority of cases (80–85%), PHPT is caused by one or more parathyroid adenoma(s) (PAs). However, PHPT can also be ascribed to hyperplasia or parathyroid gland malignancy.^4^,^5^

In the past, the “gold standard” of surgical diagnosis and treatment for PHPT was bilateral neck exploration (BNE), a very invasive procedure involving identification of all the parathyroid glands, the removal of the adenoma(s), and often the biopsy of any remaining parathyroid gland(s). BNE, while not only invasive, is a costly and lengthy procedure and can in some cases prove unsuccessful.^2^,^4^ Alternatively, the use of minimally invasive radioguided surgery (MIRS) for the treatment of PHPT, especially in patients presenting with a solitary PA, is increasing in popularity and producing quite successful results with improved patient outcomes.^4^ In fact, a survey carried out in the last decade within the International Association of Endocrine Surgeons revealed that 59% of parathyroid surgeons worldwide reported the use of some form of minimally invasive parathyroidectomy.^2^,^6^

Appropriate pre-operative imaging – in particular, parathyroid scintigraphy – is a major factor contributing to the success of MIRS. Early parathyroid imaging techniques were based on different methods of radiopharmaceutical uptake in the thyroid and parathyroid glands, making use of radiopharmaceuticals such as ^75^Selenium(^75^Se)-Methionine or ^201^Thallium(^201^Tl)-Chloride in conjunction with radioiodine and ^99m^Tc-pertechnetate.^4^ ^123^Iodine (^123^I)/^99m^Tc 2-methoxyisobutyl isonitrile (^99m^Tc MIBI) dual-tracer subtraction techniques are not preferred in Australia as ^123^I is expensive and its availability is limited. This technique might be favoured in locations where ^123^I is readily available due to the advantage of simultaneous acquisition of ^123^I and ^99m^Tc MIBI images; however, ^99m^Tc-pertechnetate is most likely to have a cost advantage. Positron emission tomography (PET) using ^18^F-fluorodeoxyglucose (^18^F-FDG) and ^11^Carbon (^11^C)-methionine to detect PAs has also been investigated in recent years, although it is not commonly used as its role in identifying parathyroid pathologies is still unknown.^4^,^7^

Today the most commonly used parathyroid scintigraphy protocols make use of a combination of ^99m^Tc-pertechnetate and ^99m^Tc MIBI, as ^99m^Tc MIBI has been found to be highly sensitive in detecting parathyroid lesions.^8^ ^99m^Tc MIBI is a lipophylic, cationic complex; its uptake is affected by many factors, including blood flow and capillary permeability, plasma and mitochondrion membrane potentials, and cellular mitochondrial contents.^8^ Originally implemented as an alternative to the perfusion tracer thallous chloride (^201^Tl) for myocardial single-photon emission computed tomography (SPECT) imaging, ^99m^Tc MIBI has also been used to assess breast lesion malignancy in patients with an abnormal mammogram or palpable mass,^9^ and in localizing distant and local foci of recurrence and metastases in patients suffering from medullary thyroid carcinoma.^10^

On intravenous injection, normal thyroid and parathyroid tissue show rapid uptake of ^99m^Tc MIBI within the first 10–20 min and then a more gradual washout over 2 h.^11^ Tissue concentration of ^99m^Tc MIBI is proportional to blood flow, gland size, and mitochondrial activity.^4^ In the absence of disease, normal parathyroid glands are not normally seen separately from the thyroid gland on the scan; however, more avid uptake and delayed clearance of ^99m^Tc MIBI from PAs results in higher lesion-to-normal tissue ratio over time, allowing for clearer visualization of PAs during delayed imaging. The difference in ^99m^Tc MIBI retention may be attributed to the presence of mitochondria-rich oxyphil cells in PAs which ^99m^Tc MIBI is known to accumulate.^3^,^11^

^99m^Tc MIBI is currently the radiopharmaceutical of choice for the intra-operative PA localization for MIRS; however, methods of its use appear to vary greatly.^12^–^14^ Currently, MIRS – despite its many benefits – has not been widely adopted.^15^ This article attempts to challenge traditional practices by examining the current literature concerning the use and efficacy of ^99m^Tc MIBI in conjunction with MIRS and bring to light the many benefits of using this technique in the treatment of PHPT. This review examines the various protocols currently in practice utilizing ^99m^Tc MIBI for MIRS, the advantages and disadvantages of MIRS for treatment of PHPT as opposed to BNE, and the improved outcomes offered not only to patients but also to staff and surgeons by this technique. By evaluating and highlighting current information, physicians and health professionals in Australia will become aware of the benefits of this innovative technique potentially increasing its use, thereby importantly improving clinical outcomes for patients suffering from PHPT.

## Methods and Materials

Using both the Embase and ProQuest databases from 2002 to 2012, approximately 40 articles were recovered using the following search terms: primary hyperthyroidism, PA, parathyroid disease, ^99m^Tc MIBI, radioguided surgery, minimally invasive parathyroidectomy, and gamma probe. Inclusion criteria for the articles reviewed were as follows: (1) the articles must be available for review in English; (2) the study must involve humans, not animals; (3) the study must involve the use of ^99m^Tc MIBI for PA localization, although studies using other radiopharmaceuticals for localization were not recovered in the last 10 years; and (4) studies must involve ^99m^Tc MIBI for use in intra-operative PA localization. Any studies using scintigraphy for investigation of suspected PA alone were not considered as this article is focusing solely on the use of ^99m^Tc MIBI to aid surgical localization and removal of PA, not diagnosis alone. The appropriateness of each article was confirmed by investigating the abstract and methods and materials section of each article. Of the articles found 27 were deemed to be relevant and in accordance with the above criteria for this literature review.

## Discussion

### Use of different protocols

Information from the last decade shows there are many different protocols in place around the world concerning the use of ^99m^Tc MIBI for pre-operative and intra-operative localization of parathyroid disease. Pre-operative scintigraphy methods include dual-tracer and/or dual-phase methods, as well as planar and SPECT protocols. Methods using ^99m^Tc MIBI for intra-operative PA localization include single-day, separate-day, and SPECT-only methods of planning.^16^,^17^

**Figure 1:**
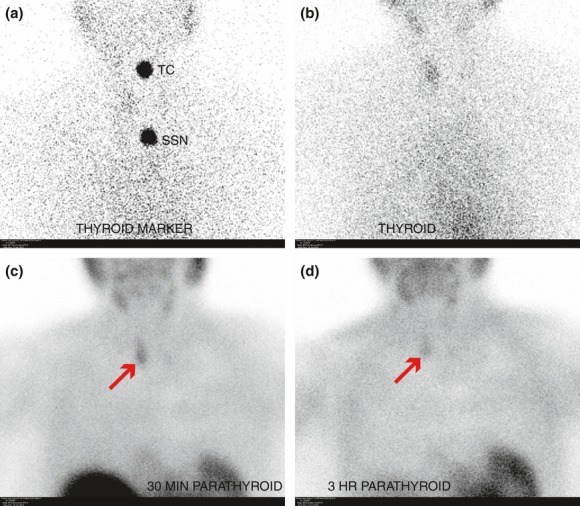
Planar parathyroid scintigraphy. Planar images demonstrating dual-tracer, dual-phase parathyroid scintigraphy using ^99m^Tc-pertechnetate and ^99m^Tc MIBI. Images (a) and (b) demonstrating the thyroid show fairly uniform uptake in the right lobe with no uptake in the left lobe of the thyroid, consistent with the patient's hemithyroidectomy. ^99m^Tc MIBI images (c) and (d) acquired at 30 min and 3 h post-injection reveal focal tracer uptake in the area of the lower pole of the right thyroid lobe, as indicated by the arrow. ^99m^Tc MIBI, technetium-99m 2-methoxyisobutyl isonitrile. Courtesy of Bankstown-Lidcombe Hospital Nuclear Medicine and Ultrasound Department.

From first reports of its use in 1989, ^99m^Tc MIBI has been the radiopharmaceutical of choice in the investigation of suspected parathyroid pathologies.^18^ Today, there are three main techniques of parathyroid scintigraphy employed in practices in Australia, single-phase dual-tracer subtraction imaging using ^99m^Tc-pertechnetate/^99m^Tc MIBI, dual-phase single-tracer imaging using ^99m^Tc-pertechnetate/^99m^Tc MIBI, and dual-tracer dual-phase imaging again using ^99m^Tc-pertechnetate/^99m^Tc MIBI with planar images acquired or a combination of planar, SPECT, or SPECT/computed tomography (SPECT/CT).^4^,^18^,^19^ Dual-tracer subtraction imaging involves the administration of ^99m^Tc MIBI for visualization of abnormal parathyroid tissue, as well as the administration of ^99m^Tc-pertechnetate for thyroid visualization. Thyroid images are then subtracted from the aforementioned ^99m^Tc MIBI parathyroid images, in order to reveal the PA.^18^ Unfortunately, artefacts on these images can be common if the patient is not in exactly the same position for both portions of the study.^18^ On the other hand, single-tracer dual-phase methods require the administration of only one radiopharmaceutical, ^99m^Tc MIBI, with images acquired at 10–15 min and 1.5–3 h post-injection. This method is based on the fact that ^99m^Tc MIBI is retained in abnormal parathyroid tissue longer than normal thyroid tissue.^18^ Dual-tracer dual-phase protocols, demonstrated by Figure 1, involve the administration of a small dose (generally 40 MBq) of ^99m^Tc-pertechnetate with thyroid gland images acquired 20 min post-injection. ^99m^Tc MIBI is administered immediately after thyroid image acquisition and additional images are acquired at 30 min and 1.5–3 h post ^99m^Tc MIBI administration. Data acquired of the thyroid during the initial ^99m^Tc-pertechnetate imaging phase is then subtracted from the ^99m^Tc MIBI images.^4^

While traditional parathyroid scintigraphy involved the acquisition of planar images alone, studies reviewed in the current literature have demonstrated the significant benefits of acquiring additional ^99m^Tc MIBI SPECT and SPECT/CT images. The increased sensitivity offered by ^99m^Tc MIBI SPECT data as opposed to planar data alone is discussed in an article by Rubello et al. The study highlights the benefits of including SPECT acquisition in pre-surgical planning for minimally invasive parathyroidectomy (MIP). In a study involving 54 PHPT patients, both dual-tracer dual-phase subtraction planar images using ^99m^Tc-pertechnetate and ^99m^Tc MIBI and additional ^99m^Tc MIBI SPECT images were acquired. Of the 54 patients, solitary PAs were identified in 87%, and two or more PAs were identified in 7.4% of patients, with an overall sensitivity of 94.6%. 12.6% of patients demonstrated a PA located deep in the para-oesophageal/para-tracheal space. It is for these patients that SPECT data prove most useful, and allow for accurate, safe, and efficient PA localization during surgery with minimal surgical trauma, which is particularly advantageous in patients who have undergone previous thyroid or parathyroid surgery.^16^,^20^,^19^

**Figure 2:**
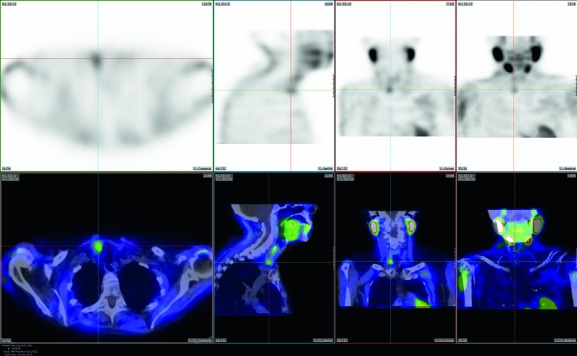
Use of SPECT/CT to aid parathyroid adenoma detection. SPECT-CT images clearly demonstrating a focal area of ^99m^Tc MIBI uptake localized in the lower pole of the right thyroid lobe, as indicated on the (a) axial, (b) coronal, and (c) sagittal imaging planes, as well as the (d) CT, (e) SPECT, and (f) fused data by an arrow. SPECT, single-photon emission computed tomography; CT, computed tomography; ^99m^Tc MIBI, technetium-99m 2-methoxyisobutyl isonitrile. Courtesy of Bankstown-Lidcombe Hospital Nuclear Medicine and Ultrasound Department.

Similarly, a study performed by Lorberboym et al. of 52 patients showed that planar scintigraphy identified 41 (79%) PAs, whereas SPECT imaging was found to be superior in 9 cases, increasing the sensitivity of the data to 96%.^21^ And again, in a separate article by Rubello, a study of 211 patients revealed deep PA in 22 patients upon investigation of SPECT images, which were confirmed surgically.^6^ An example of such imaging is included below. Figure 2 demonstrates SPECT/CT images of the parathyroid and how in this particular case these images allowed for easy detection of the PA in all planes, as well as enhanced surgical planning. One article by Schachter et al. proposed that acquisition of an early post-injection ^99m^Tc MIBI SPECT study was all that was required for pre-operative localization for MIRS for the treatment of PHPT. For the purposes of this study 82 patients suffering from PHPT underwent standard planar dual-phase dual-tracer subtraction imaging for PA localization. On the day of surgery the patient received a 740 MBq dose of ^99m^Tc MIBI for imaging and assistance with intra-operative localization of abnormal parathyroid tissue. A planar image was acquired at 10 min post-radiopharmaceutical administration followed immediately by a SPECT study. SPECT data correctly identified adenomas in 96% of cases, whereas the sensitivity of the planar data was only 78%. The SPECT data provided three-dimensional information concerning the location of the PA and was useful in 10 patients with multi-nodular goitre, three with ectopic adenomas, and two patients with two adenomas each.^16^

For the use of ^99m^Tc MIBI in MIRS, the single-day protocol involves the administration of a standard radiopharmaceutical dose of ^99m^Tc MIBI (740 MBq) followed by imaging, with surgery taking place around 3 h later the same day.^4^,^18^ In some instances the single-day protocol is favoured as it involves only one administration of radiopharmaceutical for the purpose of both imaging and intra-operative assistance; thus, the patient is not exposed to additional radiation.^4^

However, literature in the past decade favours an alternate separate-day protocol, often referred to as the “low-dose ^99m^Tc MIBI protocol.” This method, generally involves ^99m^Tc MIBI dual-tracer dual-phase subtraction scintigraphy in the days or weeks prior to MIRS, allowing more time for image acquisition and interpretation, as well as accurate selection of patients deemed eligible for MIRS.^4^,^13^,^18^ On the day of surgery, 37 MBq ^99m^Tc MIBI is administered in theatre shortly prior to commencement of the operation. There is some inconsistency in the present literature as to when ^99m^Tc MIBI should be administered. Times documented ranged from a few minutes, 10 min, and 10–30 min prior to incision.^2^,^4^,^6^ Perhaps the most constructive of these methods is the administration of 37 MBq in the few minutes prior to surgery, as the radiation exposure not only to the patient but also to the surgeon and operating staff is minimal, and importantly, potential false-negative results due to PA with reasonably fast ^99m^Tc MIBI washout can be prevented.^2^

Conversely, the review recovered two articles using a modified version of the separate-day protocol. Instead of ^99m^Tc MIBI administration a few minutes to 30 min prior to the commencement of surgery, these studies opted for the administration of 185–370 MBq and 296–370 MBq 1 h and 2 h prior to surgery, respectively.^22^,^23^ The current literature expresses the sufficiency of low-dose protocols making use of 37 MBq ^99m^Tc MIBI shortly prior to MIRS as a tenfold lower radiation dose has been proven to be adequate for the same purpose.^2^,^6^,^14^ It could be speculated that studies using the higher dose protocol may have done so due to local instrumentation requirements or the fact that injecting so close to surgery was deemed impractical to their staff, although this is not clearly outlined in the literature.

Current literature appears to heavily favour a separate-day low-dose ^99m^Tc MIBI protocol for several reasons: the delayed period of time between image acquisition and surgery allows a greater length of time for image review and interpretation by specialists; it allows for adequate surgical planning including the appropriate selection of patients who are deemed suitable for MIRS and those who may be eligible to have the procedure using local anaesthesia; and more time to plan surgery, which is particularly beneficial for efficient and productive surgical scheduling as BNE takes approximately 60 min as opposed to a 20- to 30-min procedure for MIRS.^4^,^6^,^13^ Using a separate-day protocol with the administration of 37 MBq prior to surgery also allows for an extremely low, almost negligible radiation dose to be administered to the patient, thus in turn reducing the levels of radiation exposure to the surgeon and theatre staff.^2^,^6^,^13^

Aside from these benefits, a significant advantage of using a separate-day low-dose protocol for MIRS is the increased accuracy achieved by administering the radiopharmaceutical close to intra-operative localization. In a single-day protocol, 2–3 h may have elapsed before the patient enters surgery. In these cases, ^99m^Tc MIBI may have already washed out from PAs with fast ^99m^Tc MIBI washout, reducing the intra-operative parathyroid/background and parathyroid/thyroid count ratios. This issue is of particular importance when investigating patients with nodular goitre and can be responsible for false-negative findings in surgery.^2^,^6^

### Use of MIRS for PHPT treatment

Over the last ten to fifteen years, surgical treatment of PHPT has shifted from a very invasive BNE to more limited neck explorations including unilateral neck surgery and minimally invasive radioguided parathyroidectomy. The use of neck ultrasound in conjunction with ^99m^Tc MIBI scintigraphy is imperative when planning minimally invasive forms of parathyroidectomy. Following imaging, only approximately 60–70% of patients with PHPT will be considered eligible for MIRS.

Performing MIRS for PHPT is deemed to be most appropriate for patients with a high probability of a solitary PA, considerable uptake of ^99m^Tc MIBI in the PA, no concomitant ^99m^Tc MIBI avid thyroid nodules or multi-nodular goitre, no history of familial hyperparathyroidism or multiple endocrine neoplasia, and no history of neck irradiation.^4^,^13^ In patients with multi-nodular goitre, thyroid nodules can give false-positive results during both pre-operative and intra-operative investigation due to the uptake and retention of ^99m^Tc MIBI. Use of the dual-tracer technique in these patients significantly increases the sensitivity of the scan and drastically decreases the occurrence of false-positive results.^4^ The combination of ^99m^Tc MIBI imaging with high-resolution neck ultrasound has been shown throughout the current literature to give the most effective and accurate information of solitary PA, as the combination of imaging is able to differentiate between solitary PA and multi-gland disease, while also ruling out the presence of nodular goitre.^6^

The procedure for intra-operative gamma probe use and removal of PAs was largely the same across the current literature. Prior to commencement of surgery and 10 min post-PA excision, blood samples are obtained from a peripheral vein for intra-operative quick PTH (QPTH) assay. Preceding surgical incision, the patient's neck is scanned using an 11-mm collimated gamma probe to ascertain the maximum count activity area corresponding to the previously acquired imaging.^2^,^13^,^22^,^24^,^25^ When the area of the suspected PA is identified, in vivo counts are recorded as a percentage of the background counts. A 1.5- to 2-mm incision is then made either 1 cm above the sternal notch or in the lateral neck anterior to the border of the sternocleidomastoid muscle, depending on the surgeon and the position of the PA.^24^ Incisions above the sternal notch can be easily converted into a BNE if deemed necessary.^13^ The probe is then repeatedly inserted into the incision guiding the surgeon to the area of the adenoma. It has been found in some patients with deeply located PAs that binding of the middle thyroid vein and inferior thyroid artery is sometimes required.^13^ When the adenoma has been located, radioactivity is measured along with radioactivity of the thyroid gland and background count rates. Once removed, radioactivity count rate of the PA is measured ex vivo, as well as the radioactivity count rate of the surgical bed to ensure that all abnormal parathyroid tissue has been excised.^2^,^13^,^22^,^24^,^25^

The use of ^99m^Tc MIBI for assistance in MIRS for parathyroidectomy is still a relatively new procedure. Despite positive and improved outcomes for patients undergoing MIRS for treatment of PHPT, many endocrinologists still prefer to use a more invasive, unfocussed BNE technique. An article in 2004 by Farley demonstrated that this was largely attributable to the fact that intra-operative use of gamma probes was difficult and awkward. Gamma probe accuracy based on the uptake and localization of ^99m^Tc MIBI was decreased when compared with intra-operative PTH monitoring. Also, the radiation dose to the patient, surgeon, and operating staff was a concern.^12^

Alternatively, an article by Rubello et al. in 2004 was very supportive of gamma probe assistance in minimally invasive surgery and highlighted the benefits of this technique for intra-operative localization of abnormal parathyroid tissue. First, the probe allowed for quick detection of PA intra-operatively, particularly in patients with ectopic or deep PA. Additionally, the gamma probe allowed the surgeon to easily and quickly evaluate the removal success of the hyperfunctioning parathyroid tissue by measuring residual activity in the parathyroid bed and radioactivity of the removed PA ex vivo.^2^ Upon PA removal, the count rate measured over the excised tissue should be at least 20% higher than room background. The aforementioned article by Rubello demonstrated that for all patients within the study, ex vivo radioactivity lesion-to-background count ratio was higher than 120% for all PA(s) and at least one dominant enlarged parathyroid gland in patients with multi-gland disease.^6^ Inconsistencies may be explained by the different timing of radiopharmaceutical injection and intra-operative measurement, and differences in the washout and clearance of ^99m^Tc MIBI from normal and abnormal thyroid and hyperfunctioning parathyroid tissue.^6^ In instances where inconsistencies are present, the use of QPTH assay intra-operatively can be used to confirm the successful PA removal, in which case PTH levels will be reduced by 50% or more from baseline values generally 10–30 min post-adenoma removal.^6^,^24^ In instances where PTH levels do not drop sufficiently, the presence of multi-glandular disease is highly likely.^6^,^24^,^26^

### Disadvantages of MIRS for PHPT treatment

There is limited information found in the current literature that discourages the use of MIRS for parathyroidectomy. However, some disadvantages are discussed. The main disadvantage of this technique to appear in the literature is the radiation exposure associated with radioguided surgery. Conversely, an article by Rubello et al. discusses the high sensitivity and accuracy of ^99m^Tc MIBI scintigraphy and the importance of its use in pre-operative imaging.^18^ One could argue that patients with suspected PHPT should routinely undergo ^99m^Tc MIBI imaging, and the additional administration of 37 MBq of ^99m^Tc MIBI for intra-operative PA localization is negligible.^2^ Furthermore, utilizing a single-day imaging and surgery protocol negates the need for any additional radiation exposure.^4^ The main consideration is “does the benefit of the procedure outweigh the radiation exposure risk to the patient?” The advantages of MIRS over tradition BNE, which is discussed further below, could be considered sufficient justification for this small additional exposure.

Another disadvantage highlighted in the literature was the exclusion criteria involved when selecting patients who are considered eligible for MIRS for the PHPT treatment.^18^ In all, 30–40% of patients suffering from PHPT are deemed unsuitable due to factors such as insignificant accumulation of ^99m^Tc in the suspected abnormal gland, concomitant ^99m^Tc MIBI avid thyroid nodules, multi-nodular goitre, history of familial hyperparathyroidism or multiple endocrine neoplasia, and history of neck irradiation.^4^,^13^,^18^ Patients suffering from multi-nodular goitre are at a particular disadvantage when using this technique as thyroid nodules have been shown to give false-positive results both pre- and intra-operatively due to the uptake and retention of ^99m^Tc MIBI.^4^

### Benefits and advantages of MIRS for PHPT treatment

Largely, the current literature supports the use of minimally invasive radioguided parathyroidectomy. The main advantages of MIRS when compared with traditional BNE include a reduction in cost, operating time, and patient recovery; less evidence of post-surgical hypocalcaemia, less pain, and complications; advantageous cosmetic results by allowing a small skin incision (the size of the incision documented in the data ranged 11–25 mm)^6^,^24^; the possibility of same-day discharge; and the possibility of local anaesthesia which is particularly beneficial in elderly patients^2^,^22^,^27^ and/or those patients with contraindications to general anaesthesia.^2^,^4^,^16^,^18^,^27^ It is also noteworthy to add that of the patient data contained in the literature, no patient undergoing MIRS for parathyroidectomy experienced any major surgical complications such as laryngeal nerve palsy or permanent hypoparathyroidism.^2^,^6^,^13^

The literature also mentioned the advantages of MIRS for PHPT treatment from a cost analysis point of view.^18^ A study in 2002 by Fahy et al. demonstrated through simulated clinical scenarios that the greatest cost savings as well as the lowest surgical risk were achieved when ^99m^Tc MIBI was used for intra-operative radioguidance.^28^ The main reductions in cost found by using MIRS for PHPT treatment as opposed to BNE were attributed to a reduction in theatre charges and a reduction in hospital stays to an average of 1.2 days in all studies except one study by Lal et al., which reported an average stay of 0.7 days.^2^,^6^,^24^,^27^–^30^ A study by Rubello et al. also highlights a valuable point concerning the cost of an intra-operative gamma probe and the use of ^99m^Tc MIBI. In many centres today intra-operative gamma probes are currently in use for sentinel node biopsy, negating the need to purchase one. Additionally, in departments that routinely perform myocardial perfusion imaging on a daily basis, the cost of using ^99m^Tc MIBI for separate-day, low-dose protocol using 37 MBq of ^99m^Tc MIBI is insignificant as the department could simply use some surplus ^99m^Tc MIBI during surgery.^2^ On the other hand, for departments that order pre-prepared unit doses from a centralized radiopharmacy, and departments where surgery happens to be booked on a day when there are no myocardial perfusion studies scheduled, using ^99m^Tc MIBI for MIRS would incur additional costs. Based on figures from a centralized radiopharmacy in Australia, as of November 2012, a single kit for the preparation of Technescan Sestamibi manufactured by Mallinckrodt Medical will cost $260–$320 depending on the amount purchased. In cases of purchasing a cold kit, the department may also need to purchase ^99m^Tc-pertechnetate for reconstitution. The same centralized radiopharmacy quotes $44.93 per 1 GBq of ^99m^Tc-pertechnetate. For single-day procedures at least 2 GBq would need to be added to the Technescan Sestamibi vial for reconstitution, thus incurring an additional fee of $89.86. For departments without radiopharmacy facilities, a ^99m^Tc MIBI unit dose measuring between 400 and 1000 MBq will cost between $114 and $230.^31^

An article published in 2004 by Rubello et al. detailed the success of MIRS for parathyroidectomy in the case of elderly patients, which is particularly noteworthy considering the incidence of PHPT rises with age and many countries have an ageing population.^2^ In a study containing 187 patients, 75 of who were 65 years of age or older, with a high scan probability of a solitary PA and significant uptake of ^99m^Tc MIBI in the suspected PA, with a normal thyroid, 100% of the elderly patient subgroup were treated successfully with no major surgical complications. Within the subgroup, 19 patients underwent MIRS successfully using local anaesthesia due to a concomitant disease contraindicating the use of general anaesthesia, as well as 8 patients who had undergone previous neck surgery. Compared to patients studied in the whole series, operating time and length of hospital stay were both comparable for patients in the subgroup, as well as the rate of transient hypocalcaemia post-MIRS.^2^

Of the studies reviewed, many discovered patients with deep or ectopic PAs during surgery.^2^,^6^,^16^ The aforementioned study by Rubello et al., containing 187 patients, discussed the value of the gamma probe in accurately localizing ectopic PAs in 15 patients (14 in the upper mediastinum and 1 at the carotid bifurcation) and 13 patients with PA located deep in the para-oesophageal/para-tracheal space.^2^ Additionally, in a study containing 211 participants, the use of ^99m^Tc MIBI and intra-operative gamma probe technique was key in localizing 15 ectopic PAs in the upper mediastinum, 1 ectopic gland at the carotid bifurcation, and 16 PAs located deep in the para-oesophageal/para-tracheal space.^6^

Benefits of performing MIRS for parathyroidectomy as opposed to BNE were also experienced by a surprisingly high number of patients who had previously undergone neck surgery of the thyroid and/or parathyroid glands. The main benefit of MIRS over BNE for these patients is the reduced risk of intra-operative complications. There are also associated cosmetic benefits.^2^ In a study by Rubello et al. including 34 patients with prior instances of neck surgery, MIRS was deemed to be successful in 26 patients, eight of whom were aged over 65 years.^2^ Another study achieved successful MIRS with 29 of 37 patients who had undergone previous neck surgery,^6^ and a study in 2006, again by Rubello et al. demonstrated successful treatment in 41 of 57 patients.^13^

### In the future

Of the articles reviewed, two addressed the use of new, technologically advanced handheld mobile gamma cameras to aid investigation and localization of PA in PHPT surgery.^32^,^33^ A study published in 2011 by Fujii et al. outlined the use of the eZ-SCOPE AN, a semiconductor gamma camera introduced by Anzai Medical Co., Ltd., Tokyo, Japan, as a handheld, regional imaging apparatus to navigate surgery for patients with PHPT. The study involved 11 consecutive patients with confirmed PHPT investigated by pathology. For this particular study, the eZ-SCOPE AN accurately localized the PA demonstrated on ^99m^Tc MIBI images in 100% of cases, and revealed the adenoma in question prior to skin incision, whereas ultrasound and CT only showed a PA in 63.6% and 72.7% of cases, respectively.^32^ This study suggests new emerging technologies such as the eZ-SCOPE AN may further aid localization and investigation of PA intra-operatively in the future.

Essentially, the current literature focuses on the use of ^99m^Tc MIBI for localization of solitary PA in patients with PHPT, and in most cases, patients with multi-glandular disease, and patients with two or more PA and those with thyroid carcinoma were removed from the research group. Of the articles reviewed there were two studies which aimed to include different groups to further investigate the scope and usefulness of MIRS. A report by Rubello et al. in 2006 included a study group of 336 patients, 38 of which were suffering from differentiated thyroid carcinoma with ^131^I-negative, but ^99m^Tc MIBI-positive locoregional recurrent disease to carry out radioguided extirpation of tumoural lesions.^13^ Furthermore, a study by Chen highlighted the usefulness of MIRS for the location and removal of hyperplasic glands.^25^ Further investigation of the use and outcomes of MIRS in patients normally deemed inappropriate for minimally invasive surgery would be beneficial to determine the scope of this treatment.

## Conclusion

^99m^Tc MIBI used in conjunction with MIRS in PHPT treatment is fast emerging as a superior technique to conventional BNE, in eligible patients. The current literature suggests that there is still some debate among professionals as to which the most suitable protocol is, although the literature is largely supportive of a separate-day, low-dose ^99m^Tc MIBI protocol utilizing the administration of a small amount of ^99m^Tc MIBI shortly prior to MIRS to aid in localization and removal of abnormal parathyroid tissue.

The information reviewed is largely in agreement with the extensive benefits of MIRS for parathyroidectomy as opposed to BNE. Such advantages include a reduction in operating and recovery time, reduced occurrence of post-surgical hypocalcaemia, reduced pain and complications such as laryngeal nerve palsy, improved cosmetic outcomes, and the possibility of using local anaesthesia in the elderly and those with contraindications to general anaesthesia.

Thus, the current literature is supportive of the use of ^99m^Tc MIBI in MIRS for the treatment of PHPT as a safe, efficient, and cost-effective treatment method as it offers improved patient outcomes both clinically and cosmetically.
